# Prognostic Value of Lymphovascular Invasion in Upper Urinary Tract Urothelial Carcinoma after Radical Nephroureterectomy: A Systematic Review and Meta-Analysis

**DOI:** 10.1155/2019/7386140

**Published:** 2019-09-03

**Authors:** Wen Liu, Lijiang Sun, Fengju Guan, Fangming Wang, Guiming Zhang

**Affiliations:** Department of Urology, The Affiliated Hospital of Qingdao University, Qingdao, China

## Abstract

This study was performed to identify the prognostic impact of lymphovascular invasion (LVI) in patients with upper urinary tract urothelial carcinoma (UTUC) after radical nephroureterectomy (RNU). A systematic search in PubMed, Embase, and the Cochrane Library was performed to identify relevant studies. The outcomes of interest, including progression-free survival (PFS), cancer-specific survival (CSS), and overall survival (OS), were extracted, and the pooled hazard ratios (HRs) and 95% confidence intervals (CIs) were used for effect size estimation. Subgroup, metaregression, and sensitivity analyses were performed to explore potential origins of heterogeneity. Publication bias was estimated by Egger's linear regression and funnel plot. Our meta-analysis included a total of 27 studies involving 17,453 patients. The pooled HRs were statistically significant for PFS (HR = 1.73, 95%CI = 1.41–2.11), CSS (HR = 1.87, 95%CI = 1.54–2.27), and OS (HR = 1.56, 95%CI = 1.29–1.87), with high heterogeneity (*I*^2^ = 77.8%, 70.3%, and 59.2%, respectively). Four studies explored the prognostic value of LVI in patients with advanced tumor stages (T3–T4). The fixed effects model (*I*^2^ = 33.9%) showed that the pooled HR was 1.64 (95%CI = 1.35–1.99) for CSS. Egger's plots showed no significant publication bias (PFS: *P* = 0.443, CSS: *P* = 0.096, and OS: *P* = 0.894). Our meta-analysis demonstrated that LVI is a poor prognostic factor for UTUC and is strongly associated with disease recurrence, cancer-specific mortality, and overall mortality.

## 1. Introduction

Upper urinary tract urothelial carcinoma (UTUC) accounts for 10% of renal tumors and 5% of all urothelial malignancies [1, 2]. Radical nephroureterectomy (RNU) with the removal of the bladder cuff is the standard treatment of UTUC, including high-risk noninvasive and invasive UTUC [3, 4]. The incidence of invasive UTUC (approximately 60%) is much higher than that of bladder cancer. The prognosis of UTUC is poor worldwide, with a recurrence rate ranging from 30% to 75% [2, 5]. Therefore, an exploration of the potential prognostic factors in UTUC is important for risk classification. Many studies have indicated that older age, a history of bladder cancer, a higher tumor stage, a higher tumor grade, lymph node metastasis, multifocality, and hydronephrosis are predictors of disease recurrence or survival [1, 5, 6].

Lymphovascular invasion (LVI) is defined as the invasion of tumor cells into an endothelium-lined space of vascular or lymphatic vessels without underlying muscular walls [7]. The process of LVI is a crucial phase in the systemic dissemination of cancer cells [8]. In cancers of the liver, testis, and penis [9], LVI is included in the American Joint Committee on Cancer (AJCC) tumor, node, metastasis (TNM) staging criteria for higher-risk patients, indicating that LVI might have a similar significance in the TNM classification. Many studies have estimated the prognostic influence of LVI in patients with UTUC, but the results remain controversial [10–36]. The European Association of Urology Guidelines indicate that LVI is an independent prognostic predictor of UTUC based on two retrospective studies [1]. One recent meta-analysis analyzed the prognostic value of LVI in UTUC but showed high heterogeneity [37]. Considering the new articles published in the past 5 years, we aimed to develop more stringent inclusion and exclusion criteria with which to further validate the prognostic impact of LVI on UTUC and explore the potential factors causing heterogeneity.

## 2. Methods

### 2.1. Literature Search

We searched several electronic databases (PubMed, Embase, Web of Science, and the Cochrane Library) for relevant studies up to 31 December 2018. The following search terms were used to identify studies focusing on the prognostic value of LVI in UTUC: (1) “upper urinary tract” and “carcinoma” or “cancer” and “lymphovascular invasion”; (2) “upper urinary tract” and “carcinoma” or “cancer” and “survival” or “Cox” or “multivariable.”

### 2.2. Study Selection

We defined the inclusion and exclusion criteria before searching for articles. Studies were included if they met the following criteria: (1) the study evaluated LVI as a prognostic factor in patients with UTUC after RNU; (2) the study reported adjusted hazard ratios (HRs) with 95% confidence intervals (CIs) of progression-free survival (PFS), cancer-specific survival (CSS), or overall survival (OS) in a multivariate analysis with Cox proportional hazard regression; and (3) the study was published in English. The exclusion criteria were as follows: (1) the study did not provide sufficient data to acquire the HR and its standard error, (2) the article described a review or study on cell lines or animal models, (3) the number of cases was <100, (4) surgical treatment was not limited to RNU, and (5) neoadjuvant chemotherapy was applied to the participants. When more than one article was based on the same study population, we included the most complete or the most recent study.

### 2.3. Endpoints and Data Extraction

The endpoints of our study were PFS, CSS, and OS. Disease recurrence was defined as local failure or distant metastasis after surgery. CSS included only patients who died of UTUC, and OS included all causes of deaths. The extracted items were as follows: first author, publication year, region, recruitment period, number of cases, definition of LVI, LVI percentage, inclusion and exclusion criteria, definition of recurrence, and adjusted covariates (age, sex, operation, tumor location, lymph node status, history of bladder cancer, adjuvant chemotherapy, primary tumor stage, tumor grade, carcinoma *in situ*, margin status, hydronephrosis, multifocality, tumor architecture, body mass index, and adjusted HR with 95% CI of PFS, CSS, and OS). Two reviewers investigated relevant articles and extracted data, respectively. Disagreements between the two reviewers regarding data abstraction were resolved through discussion.

### 2.4. Statistical Analysis

The effect measures for the outcomes of PFS, CSS, and OS were the HRs and 95% CIs in the multivariable analysis with Cox proportional hazard regression, which were extracted from all included studies. LVI was considered an independent predictor if the pooled 95% CI did not overlap with 1 and *P* < 0.05. The *I*^2^ statistic (total percentage of variation resulting from heterogeneity) was calculated to quantify the degree of heterogeneity. A fixed effects model was adopted to pool the HRs if *I*^2^ was ≤50%; otherwise, a random effects model was selected. We performed a subgroup analysis and a meta-regression analysis to explore potential heterogeneity. Next, a Galbraith plot was designed and a leave-one-out analysis was performed to search for studies causing heterogeneity and examine the weight of their influence on the pooled HR [38]. An influence analysis, in which one study was omitted and then the remaining studies were recalculated, was conducted to confirm the stability of the results. Publication bias was estimated by Egger's linear regression and funnel plot. All reported *P* values were two-sided, and the statistical significance was set at *P* ≤ 0.05. Statistical analysis was performed using Stata version 15.0 (Stata Corp., College Station, TX).

## 3. Results

### 3.1. Study Selection

**Figure 1:**
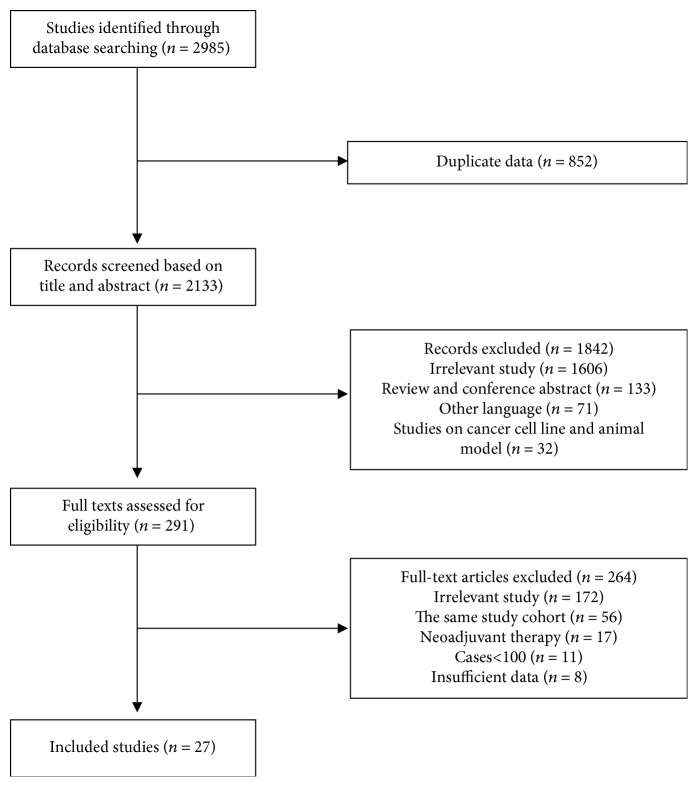
Flow chart for the identification of relevant articles.

The process of identifying relevant studies is illustrated in Figure 1. In total, 2985 articles were obtained from PubMed, Embase, and the Cochrane Library. After excluding 852 duplicate articles, we read 2133 titles and abstracts for further screening. Based on the information obtained from 291 original articles, 56 studies were excluded due to the presence of identical cohorts. Finally, 27 articles were included in this study to explore the prognostic value of LVI in UTUC [10–36], among which 4 articles analyzed LVI as a survival predictor in patients with advanced tumor stages (T3–T4) [10, 13, 16, 26].

### 3.2. Study Characteristics

Tables 1 and 2 summarize the characteristics of the 27 eligible studies. These 27 retrospective studies involved a total of 17,453 patients, with a wide recruitment period from 1987 to 2016. The number of male and female participants was 10,418 and 5320, respectively. Among the 27 studies, 9 assessed patients from Japan, 7 from China, 5 from Korea, 4 from Europe, and 2 from multiple countries. In total, 4217 patients with positive LVI were enrolled in our study, and the overall proportion of LVI was 24.2%. Studies including fewer than 100 patients were removed to limit heterogeneity; thus, the sample size of the enrolled studies ranged from 109 to 2492. Bladder recurrence was not considered a disease recurrence except in one study [18]. Twenty-two studies were adjusted for more than four covariates, and all studies adjusted for the effect of tumor stage and/or grade in the multivariable analysis.

### 3.3. Prognostic Value of LVI in UTUC

**Figure 2:**
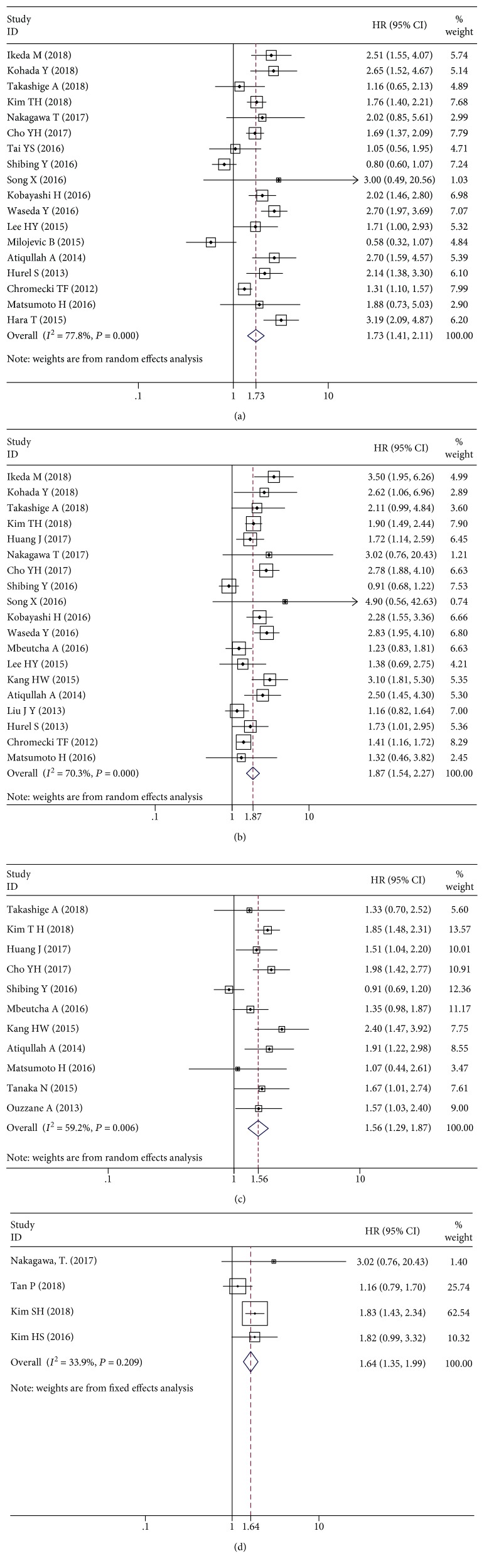
Forest plots of hazard ratios (HRs) for the association between lymphovascular invasion and upper urinary tract urothelial carcinoma. (a) Progression-free survival (PFS). (b) Cancer-specific survival (CSS). (c) Overall survival (OS). (d) Lymphovascular invasion and CSS in patients with advanced tumor stages (T3–T4).

**Figure 3:**
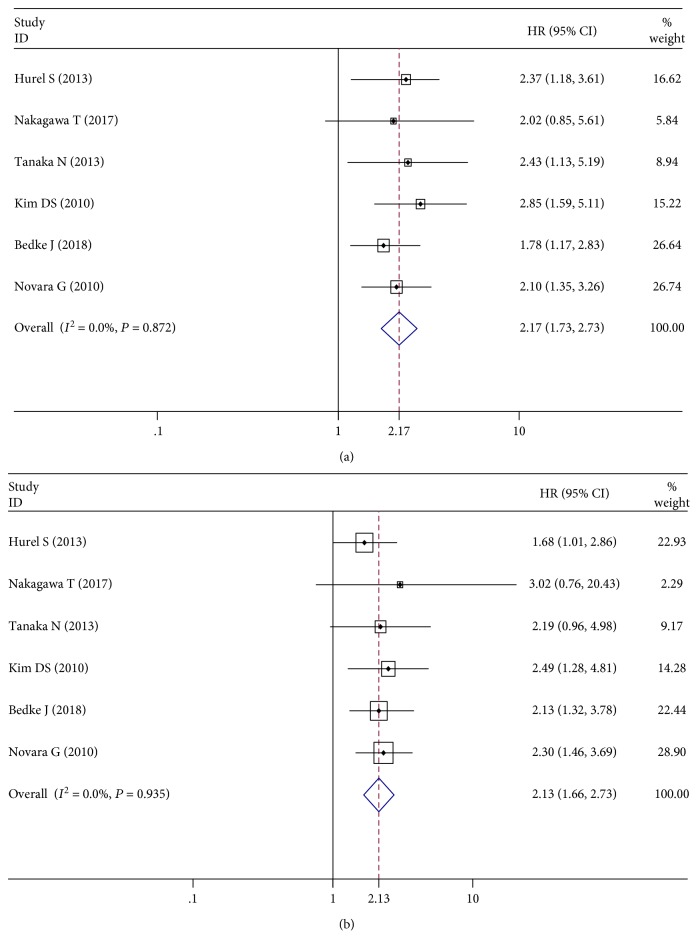
Forest plots of hazard ratios (HRs) for the association between lymphovascular invasion and upper urinary tract urothelial carcinoma in node-negative patients. (a) Progression-free survival (PFS). (b) Cancer-specific survival (CSS).

When *I*^2^ > 50%, we used a random effects model to pool the HRs. The pooled HR was statistically significant for PFS (HR = 1.73, 95%CI = 1.41–2.11), CSS (HR = 1.87, 95%CI = 1.54–2.27), and OS (HR = 1.56, 95%CI = 1.29–1.87), with high heterogeneity (*I*^2^ = 77.8%, 70.3%, and 59.2%, respectively) (shown in Figures 2(a)–2(c)). Four studies explored the prognostic influence of LVI in patients with advanced tumor stages (T3–T4). Through the fixed effects model (*I*^2^ = 33.9%), the pooled HR was 1.64 (95%CI = 1.35–1.99) for CSS (Figure 2(d)). We encompassed 6 studies that analyzed LVI as a survival predictor restricted to patients without lymph node metastasis and to those who have not undergone lymphadenectomy [16, 35, 39–42]. LVI was positively associated with the higher risk of disease recurrence (PFS: HR = 2.17, 95%CI = 1.73–2.73) and cancer-specific mortality (CSS: HR = 2.13, 95%CI = 1.66–2.73), without heterogeneity (both *I*^2^ = 0%) (Figures 3(a) and 3(b)).

### 3.4. Subgroup Analysis and Sensitivity Analysis

**Figure 4:**
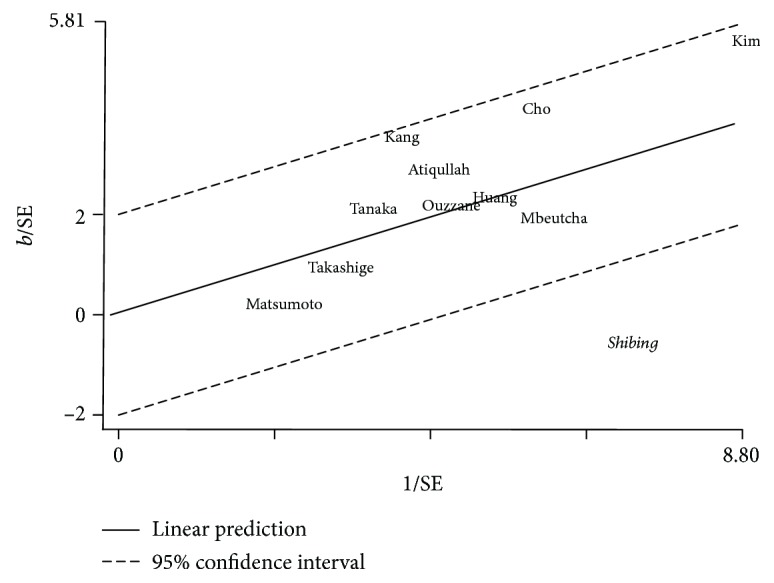
Galbraith plot showing the potential heterogeneity for overall survival. b: regression coefficient; SE: standard error.

To explore the source of potential heterogeneity, we classified the enrolled studies into those from Japan, Korea, China, and other regions (two from France, two from multiple regions, one from Germany, and one from Serbia). In PFS and CSS, the *I*^2^ value decreased in groups of Japan, South Korea, and China (Table 3). The metaregression analysis showed that the performance of the studies in different regions might have been the origin of potential heterogeneity in PFS (*P* = 0.055) and CSS (*P* = 0.011). The Galbraith plot showed that one study revealed heterogeneity in OS (Figure 4); in the leave-one-out analysis, the heterogeneity decreased to 0.0% after excluding this study [22]. The sensitivity analysis showed that the pooled HRs of PFS, CSS, and OS were stable and that none of the studies could powerfully change the positive outcome. After omitting four studies in PFS and three in CSS by the leave-one-out analysis, a stable positive result (PFS: HR = 1.84, 95%CI = 1.65–2.06; CSS: HR = 2.23, 95%CI = 1.97–2.53) indicated the prognostic value of LVI in UTUC, with low heterogeneity (PFS: *I*^2^ = 4.4%; CSS: *I*^2^ = 2.2%).

### 3.5. Publication Bias

**Figure 5:**
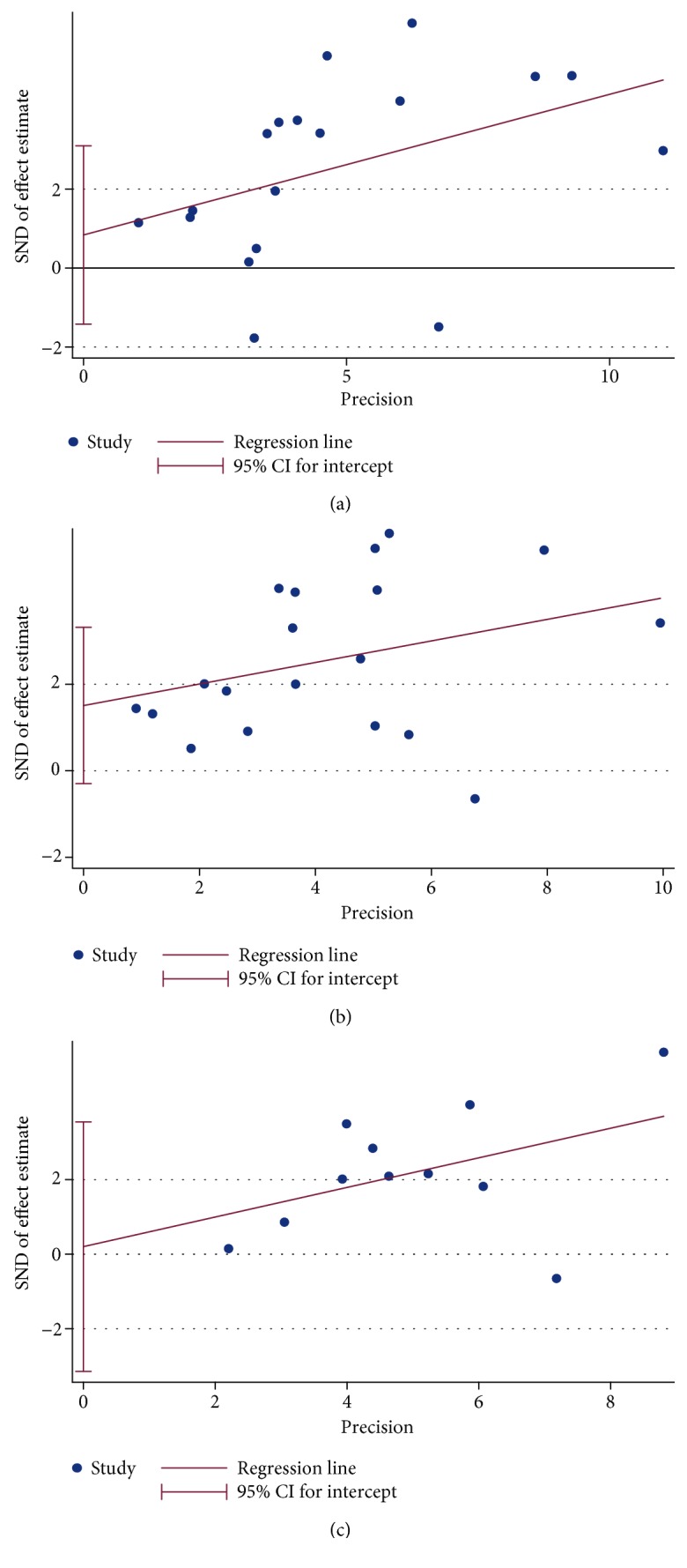
Egger's funnel plots for publication bias. (a) Progression-free survival (PFS). (b) Cancer-specific survival (CSS). (c) Overall survival (OS). SND: standard normal deviate; CI: confidence interval.

Egger's plot showed no significant evidence of publication bias for any of the three outcomes (PFS: *P* = 0.443, CSS: *P* = 0.096, and OS: *P* = 0.894) (Figures 5(a)–5(c)). Likewise, the funnel plots showed no significant evidence of publication bias (data not shown).

## 4. Discussion

Because of the poor prognosis of UTUC, it is necessary to identify high-risk patients for individual therapy. LVI is included in the AJCC TNM staging criteria for cancers of the liver, testis, and penis [9]; in these staging criteria, LVI can upstage the cancer. Although LVI was shown to increase the prognostic risk of UTUC in many studies [29–33], it is not required for stage grouping in the AJCC cancer staging manual. The present study builds on a previous meta-analysis by including recently published studies that focus on the prognostic impact of LVI on UTUC [37]. Using more stringent inclusion and exclusion criteria, we aimed to further validate the prognostic risk of LVI and explore the potential factors causing heterogeneity.

In this meta-analysis, we identified 18 studies evaluating PFS, 19 evaluating CSS, 11 evaluating OS, 4 evaluating the CSS of patients with advanced tumor stages, and 6 evaluating the PFS and CSS in node-negative patients. Given the rarity of UTUC, it is notable that the present meta-analysis has a higher sample size than that of a previously published meta-analysis [37] and has the largest amount of data. Compared with patients with UTUC and no LVI, patients with concurrent UTUC and LVI had a 1.73-fold higher risk of developing disease recurrence (HR = 1.73, 95%CI = 1.41–2.11) and a 1.87-fold higher risk of cancer-specific death (HR = 1.87, 95%CI = 1.54–2.27), suggesting that LVI is an independent prognostic predictor in patients with UTUC. These results are congruent with those of a previous meta-analysis that reported a positive association of LVI with oncologic outcomes, with a pooled HR of 1.91 and 1.72 for PFS and CSS, respectively [37]. Ku et al. did not prove that LVI was an independent factor in UTUC based on two studies (HR = 4.05, 95%CI = −0.44 to 8.53) [37]. However, LVI was evaluated as a strong predictor of poor OS in patients with UTUC (HR = 1.56, 95%CI = 1.29–1.87) through pooling of 11 studies. Only four studies explored the prognostic value of LVI in patients with advanced tumor stages (T3–T4). Through our fixed effects model (*I*^2^ = 33.9%), the pooled HR showed that LVI could increase the risk of cancer-specific death (HR = 1.64, 95%CI = 1.35–1.99). Unfortunately, most articles did not investigate the relationship between LVI and different stages of UTUC; thus, adding LVI as a predictor to TNM staging requires studies that focus on stratification of the tumor stage. Novara et al. have investigated the interaction between LVI and lymph node stage [40]. They found that LVI was associated with PFS and CSS in patients with negative lymph nodes, not in lymph node-positive patients. In our study, we revealed that the presence of LVI increased the risk of both disease recurrence and cancer-specific mortality in patients with pN0 and pNx disease (HR = 2.17 and 2.13, respectively). There was no sufficient data for us to explore the effect of LVI on node-positive patients. LVI was an essential and important step in the systemic dissemination of cancer cells [40]. Although lymph node metastasis at the time of RNU could be a reliable prognostic predictor of recurrence, no standard guideline for the appropriate area of lymphadenectomy during RNU has been established [35]. Therefore, LVI could identify patients without lymph node involvement or undergoing lymph node resection who are at an increased risk of cancer recurrence and mortality. Our study represents a more comprehensive analysis of the effect of LVI on UTUC and prompts both urologists and oncologists to select patients at a higher risk of recurrence or death because such patients may be candidates for further therapy (adjuvant chemotherapy) or more intense follow-up. On this basis, pathologists should routinely perform an exhaustive pathological assessment of UTUC specimens to identify LVI [43].

We developed more stringent exclusion criteria and conducted a subgroup analysis to decrease the clinical heterogeneity. Studies with fewer than 100 patients were excluded to rule out interference with the outcomes [43]. Studies that included neoadjuvant chemotherapy or conservative surgery, such as segmental ureterectomy with termino-terminal anastomosis, were omitted to maintain the analysis of only a single treatment (RNU) with adjuvant chemotherapy. The utility of neoadjuvant chemotherapy in patients with UTUC remains uncertain, and additional trials are thus needed; however, adjuvant chemotherapy can increase CSS and OS in patients with UTUC [44]. After the studies had been classified by region, the heterogeneity of PFS and CSS was significantly reduced except in China. For further analysis, we designed a Galbraith plot and performed a leave-one-out analysis and identified one study from China causing heterogeneity in all three outcomes [22]. This might have been due to the low percentage (11%) of LVI, which was diagnosed depending on the level of pathologists. Nonetheless, the pooled HRs of each group in the three outcomes were still positive, excluding the study from China; therefore, we considered that LVI was an independent prognostic predictor in UTUC despite the high heterogeneity. There was no heterogeneity between LVI and lymph node-negative patients, indicating the strongly predictive value of LVI on them.

The biological mechanisms of the positive association between LVI and poor UTUC outcomes are complex. LVI is defined as the presence of tumor cells within an endothelium-lined space without underlying muscular walls [7] and is an important step in tumor dissemination. Tumor cells enter the circulation through the lymphatic and blood vessels, forming micrometastases [45]. Additionally, LVI has been linked with lymph node involvement and is suggested to be a prerequisite for lymph node invasion [35, 40, 45]. Lymph node metastasis is included in the TNM classification. More importantly, the presence of LVI is associated with disease recurrence or mortality in patients with node-negative UTUC, but not in those with node-positive UTUC [40, 45]. Moreover, LVI significantly increases the risk of disease recurrence, cancer-specific mortality, and overall mortality after effective local treatment (RNU). Based on these above-described factors, LVI is considered to play an important role in the metastatic process and promote a poor prognosis [45].

To the best of our knowledge, this meta-analysis is the most comprehensive of its kind to identify the association between LVI and poor outcomes of UTUC. This meta-analysis has several strengths. First, we adopted the largest sample size (17,453 patients), although we used more stringent inclusion and exclusion criteria. This massive study population enhances the statistical power and ensures more accurate risk estimation. Second, this is the first analysis of the influence of LVI in patients with advanced tumor stages (T3–T4) and lymph node-negative UTUC through a meta-analysis with low heterogeneity. Third, no publication bias existed in our study. Our meta-analysis also had several limitations. First, all enrolled articles were retrospective studies; thus, many confounding factors could not be corrected because of a lack of patient information. For this reason, the outcomes of our meta-analysis might deviate from the actual situation in the clinical setting. Second, significant heterogeneity was found for PFS, CSS, and OS. However, we found the potential origin of heterogeneity and the article causing heterogeneity through subgroup, meta-regression, and leave-one-out analyses. After excluding the article from China, the *I*^2^ of PFS, CSS, and OS decreased to 68%, 58%, and 0%, respectively. The heterogeneity mainly came from the geographical region (for PFS and CSS). Although a random effects model was used and many statistical methods were implemented, the conclusions reached in our meta-analysis should be interpreted with caution.

## 5. Conclusions

In conclusion, the pooled results demonstrate that LVI is a significant prognostic factor for UTUC, especially for lymph node-negative patients, and is strongly associated with disease recurrence, cancer-specific mortality, and overall mortality. It can also denote a poor prognosis of UTUC with an advanced tumor stage (T3–T4). We advocate systematic assessment of LVI after RNU using pathological specimens. The results of this meta-analysis need to be further confirmed by adequately designed prospective studies before LVI is included in the AJCC TNM staging system of UTUC.

## Figures and Tables

**Table 1:** Characteristics of studies included in meta-analysis.

Study	Author	Country	Multicenter	No. of cases	Recruitment period	Exclude BC patients	Definition of LVI	No. of positive LVI (%)	Age (year)	Gender (male/female)	Definition of recurrence
2018	Ikeda et al.	Japan	Yes	441	1990-2015	No	Yes	156 (37)	69.0 (62-75)	319/122	Excluding bladder recurrence
2018	Kohada et al.	Japan	No	148	1999-2016	No	No	55 (37)	71.0 (64-78)	112/36	Excluding bladder recurrence
2018	Abe et al.	Japan	Yes	214	2000-2015	Yes	No	96 (45)	70.5 (35-93)	151/63	Excluding bladder recurrence
2018	Kim et al.	Korea	Yes	1521	2000-2012	Yes	No	332 (22)	65.0 (57-72)	1127/394	Excluding bladder recurrence
2018	Tan et al.	China	No	710	2013-2016	No	No	99 (15)	65.8	380/288	Excluding bladder recurrence
2018	Kim et al.	Korea	Yes	1276	2000-2012	Yes	No	258 (20)	65.7	941/335	Excluding bladder recurrence
2017	Huang et al.	China	No	481	2003-2013	Yes	No	76 (16)	65.8 (30-89)	311/170	NA
2017	Nakagawa et al.	Japan	Yes	109	1996-2013	Yes	No	78 (72)	71.0 (64-77)	67/42	Excluding bladder recurrence
2017	Cho et al.	Korea	Yes	1049	2004-2015	Yes	No	202 (19)	68.5	759/290	Including bladder recurrence
2016	Tai et al.	China	No	503	1996-2009	Yes	No	87 (18)	68.7 (59-75)	249/234	Excluding bladder recurrence
2016	Shibing et al.	China	Yes	795	2002-2012	Yes	Yes	84 (11)	NA	462/333	Excluding bladder recurrence
2016	Song et al.	China	No	140	2005-2011	Yes	No	6 (4)	NA	86/54	Excluding bladder recurrence
2016	Kobayashi et al.	Japan	Yes	839	1990-2011	Yes	Yes	326 (39)	70.4 (63-78)	610/229	Excluding bladder recurrence
2016	Waseda et al.	Japan	Yes	1068	1995-2013	Yes	No	446 (42)	70.0 (62-76)	758/310	Excluding bladder recurrence
2016	Mbeutcha et al.	International	Yes	716	1990-2008	No	No	149 (21)	70.0 (63-76)	400/316	NA
2016	Kim et al.	Korea	No	371	1992-2012	Yes	Yes	71 (19)	64.7	287/84	NA
2016	Matsumoto et al.	Japan	No	144	1995-2010	No	No	93 (65)	71.0 (14-98)	104/40	NA
2015	Hara et al.	Japan	Yes	1172	2005-2011	No	Yes	423 (36)	71.0 (21-97)	806/366	Excluding bladder recurrence
2015	Tanaka et al.	Japan	Yes	394	1995-2011	Yes	Yes	170 (43.1)	70.0	289/105	Excluding bladder recurrence
2015	Lee et al.	China	No	250	2004-2010	Yes	Yes	60 (24)	NA	108/142	Excluding bladder recurrence
2015	Kang et al.	Korea	Yes	440	2001-2013	Yes	No	76 (17)	NA	305/135	Excluding bladder recurrence
2015	Milojevic et al.	Serbia	No	238	1999-2013	Yes	No	154 (65)	66.5	132/106	Excluding bladder recurrence
2014	Aziz et al.	Germany	No	256	1990-2012	Yes	Yes	52 (20)	68.6	169/96	Excluding bladder recurrence
2013	Liu et al.	China	Yes	421	1999-2010	Yes	Yes	101 (24)	NA	285/136	NA
2013	Hurel et al.	France	Yes	551	1995-2010	Yes	Yes	163 (30)	69.4	365/188	Excluding bladder recurrence
2013	Ouzzane et al.	France	Yes	714	1995-2010	Yes	No	157 (22)	70.0	484/228	Excluding bladder recurrence
2012	Chromecki et al.	International	Yes	2492	1987-2007	Yes	Yes	247 (10)	69.2	1681/811	Excluding bladder recurrence

NA: not available; BC: bladder cancer; LVI: lymphovascular invasion.

**Table 2:** Outcomes of studies included in meta-analysis.

Year	Author	PFS HR (95% CI)	CSS HR (95% CI)	OS HR (95% CI)	No. of covariates	Adjusted variables (part)
2018	Ikeda et al.	2.51 (1.552-4.07)	3.5 (1.954-6.259)	NA	11	Age, gender, tumor location, pN, history of BC, adjuvant therapy, pT, tumor grade, CIS, tumor architecture
2018	Kohada et al.	2.65 (1.52-4.67)	2.62 (1.06-6.96)	NA	5	pT, tumor grade, hydronephrosis
2018	Abe et al.	1.162 (0.647-2.133)	2.11 (0.988-4.839)	1.326 (0.697-2.52)	3	pN, pT, tumor grade
2018	Kim et al.	1.76 (1.4-2.21)	1.9 (1.49-2.44)	1.85 (1.48-2.31)	14	Age, gender, operation, tumor location, pN, history of BC, adjuvant therapy, pT, tumor grade, CIS, BMI
2018	Tan et al.	1.02 (0.73-1.43)	1.16 (0.79-1.7)	1.16 (0.82-1.65)	14	Age, gender, tumor location, pN, adjuvant therapy, pT, tumor grade, tumor architecture, multifocality
2018	Kim et al.	1.859 (1.464-2.36)	1.829 (1.43-2.338)	1.776 (1.36-2.32)	8	Tumor grade, tumor architecture, margin status, pN
2017	Huang et al.	NA	1.72 (1.14-2.59)	1.51 (1.04-2.2)	6	Age, pN, pT, tumor grade, multifocality
2017	Nakagawa et al.	2.02 (0.85-5.61)	3.02 (0.76-20.43)	NA	5	Age, adjuvant therapy, tumor grade, hydronephrosis
2017	Cho et al.	1.69 (1.37-2.09)	2.78 (1.88-4.1)	1.98 (1.42-2.77)	19	Age, gender, operation, tumor location, pN, history of BC, pT, CIS, hydronephrosis, multifocality, BMI
2016	Tai et al.	1.05 (0.56-1.95)	NA	NA	3	Tumor location, pT, tumor grade
2016	Shibing et al.	0.802 (0.6-1.072)	0.909 (0.68-1.215)	0.913 (0.695-1.2)	10	Age, operation, tumor location, pN, pT, tumor grade, tumor architecture
2016	Song et al.	3.165 (0.487-20.565)	4.898 (0.563-42.626)	NA	15	Age, gender, tumor location, history of BC, pT, tumor grade, hydronephrosis
2016	Kobayashi et al.	2.02 (1.46-2.8)	2.28 (1.55-3.36)	NA	6	Age, gender, pN, adjuvant therapy, pT, tumor grade
2016	Waseda et al.	2.7 (1.97-3.69)	2.83 (1.95-4.1)	NA	5	Age, tumor location, pN, pT, tumor grade
2016	Mbeutcha et al.	NA	1.23 (0.83-1.81)	1.35 (0.98-1.87)	12	Age, gender, tumor location, pN, history of BC, adjuvant therapy, pT, tumor grade, CIS, tumor architecture
2016	Matsumoto et al.	1.88 (0.73-5.03)	1.32 (0.46-3.82)	1.07 (0.44-2.61)	6	pT, tumor grade
2016	Kim et al.	NA	1.82 (0.99-3.32)	NA	3	Tumor location, history of BC, tumor grade
2015	Tanaka et al.	NA	NA	1.67 (1.01-2.74)	4	Age, adjuvant therapy, pT, tumor grade
2015	Hara et al.	3.19 (2.09-4.87)	NA	NA	16	Age, gender, operation, tumor location, pN, history of BC, adjuvant therapy, pT, tumor grade, margin status
2015	Lee et al.	1.71 (1-2.93)	1.38 (0.69-2.75)	NA	4	pN, pT, tumor grade
2015	Kang et al.	NA	3.097 (1.81-5.297)	2.4 (1.469-3.921)	9	Age, adjuvant therapy, pT, tumor grade, margin status, multifocality, adjuvant therapy, BMI
2015	Milojevic et al.	0.58 (0.32-1.07)	NA	NA	5	pN, history of BC, adjuvant therapy, pT, tumor grade
2014	Aziz et al.	2.7 (1.59-4.57)	2.5 (1.45-4.3)	1.91 (1.22-2.98)	10	Age, tumor location, pN, pT, tumor grade, tumor architecture, multifocality
2013	Liu et al.	NA	1.16 (0.818-1.645)	NA	13	Age, gender, operation, tumor location, pN, adjuvant therapy, pT, tumor grade, margin status, multifocality
2013	Hurel et al.	2.14 (1.38-3.3)	1.73 (1.01-2.95)	NA	8	Age, tumor location, pN, adjuvant therapy, pT, tumor grade, margin status
2013	Ouzzane et al.	NA	NA	1.57 (1.03-2.4)	6	Age, tumor location, pN, pT, tumor grade, margin status
2012	Chromecki et al.	1.31 (1.1-1.57)	1.41 (1.16-1.72)	NA	10	Age, gender, tumor location, pN, adjuvant therapy, pT, tumor grade, tumor architecture, multifocality

NA: not available; BC: bladder cancer; PFS: progression-free survival; CSS: cancer-specific survival; OS: overall survival; HR: hazard ratio; CIS: carcinoma in situ; pT: pathological tumor stage; pN: pathological lymph node.

**Table 3:** Subgroup analyses by region and meta-regression analyses for progression-free survival, cancer-specific survival, and overall survival.

Region	No. of studies	Weight (%)	OR (95% CI)	*I* ^2^	*P* for heterogeneity	*P* ^∗^ for interaction
PFS	18	100.00	1.73 (1.41-2.11)	77.8%	<0.001	0.055
Japan	8	41.91	2.32 (1.90-2.83)	26.3%	0.219	
Korea	2	15.47	1.72 (1.47-2.01)	0.0%	0.798	
China	4	18.30	1.15 (0.72-1.84)	59.3%	0.061	
Others^a^	4	24.32	1.47 (0.89-2.43)	83.6%	<0.001	
CSS	19	100.00	1.87 (1.54-2.27)	70.3%	<0.001	0.011
Japan	7	28.60	2.57 (2.06-3.20)	0.0%	0.743	
Korea	3	19.89	2.40 (1.74-3.29)	53.3%	0.117	
China	5	25.94	1.25 (0.92-1.69)	51.2%	0.085	
Others ^a^	4	25.57	1.54 (1.20-1.97)	39.9%	0.172	
OS	11	100.00	1.56 (1.29-1.87)	59.2%	<0.001	0.636
Japan	3	16.68	1.44 (1.01-2.07)	0.0%	0.661	
Korea	3	32.24	1.95 (1.64-2.31)	0.0%	0.635	
China	2	22.37	1.15 (0.71-1.89)	77.9%	0.033	
Others^a^	3	28.72	1.53 (1.23-1.92)	0.0%	0.463	

^∗^ *P* values for meta-regression. ^a^Two studies from France, two from multiple countries, one from German, and one from Serbia. PFS: progression-free survival; CSS: cancer-specific survival; OS: overall survival.
